# Inhibition of Rice Serotonin *N*-Acetyltransferases by MG149 Decreased Melatonin Synthesis in Rice Seedlings

**DOI:** 10.3390/biom11050658

**Published:** 2021-04-29

**Authors:** Kyungjin Lee, Geun-Hee Choi, Kyoungwhan Back

**Affiliations:** 1 Department of Biotechnology, College of Agriculture and Life Sciences, Chonnam National University, Gwangju 61186, Korea; nicekj7@hanmail.net; 2 Nakdonggang National Institute of Biological Resources, 137, Donam 2-gil, Sangju-si 37242, Korea; ghchoi@nnibr.re.kr

**Keywords:** histone acetyltransferase inhibitor, melatonin, MB3, MG149, rice, serotonin *N*-acetyltransferase

## Abstract

We examined the effects of two histone acetyltransferase (HAT) inhibitors on the activity of rice serotonin *N*-acetyltransferases (SNAT). Two rice recombinant SNAT isoenzymes (SNAT1 and SNAT2) were incubated in the presence of either MG149 or MB3, HAT inhibitors. MG149 significantly inhibited the SNAT enzymes in a dose-dependent manner, especially SNAT1, while SNAT2 was moderately inhibited. By contrast, MB3 had no effect on SNAT1 or SNAT2. The application of 100 μM MG149 to rice seedlings decreased melatonin by 1.6-fold compared to the control, whereas MB3 treatment did not alter the melatonin level. MG149 significantly decreased both melatonin and *N*-acetylserotonin when rice seedlings were challenged with cadmium, a potent elicitor of melatonin synthesis in rice. Although MG149 inhibited melatonin synthesis in rice seedlings, no melatonin deficiency-induced lamina angle decrease was observed due to the insufficient suppression of SNAT2, which is responsible for the lamina angle decrease in rice.

## 1. Introduction

Melatonin is a signaling molecule and potent antioxidant in plants [1,2,3,4]. It has diverse physiological roles in plant growth and development, affecting seed longevity [5], germination [6], seedling growth [7], flowering [8], seed yield [9], photosynthesis [10,11], endoplasmic reticulum quality [12,13], and secondary metabolite biosynthesis [14,15]. Melatonin is also closely involved in the plant defense response to biotic and abiotic stresses, including pathogens and heavy metals [16,17,18]. These biological effects may require a melatonin receptor [19,20] and signaling cascade, such as a mitogen-activated protein kinase [4], although melatonin exerts its antioxidant activity against oxidative stress directly via a receptor-independent pathway [21,22].

The pleiotropic effects of melatonin in plants have been characterized using either exogenous melatonin treatment or molecular genetic analyses following the successful cloning of melatonin biosynthesis and metabolism genes [4]. However, the high catalytic efficiency of melatonin degradation enzymes results in very low levels of endogenous melatonin in plants. This often causes difficulty proving the endogenous functions of melatonin via gain- and loss-of-function analyses of related genes [4]. To compensate for the weakness of molecular genetic analyses in melatonin studies in plants, it is necessary to use a specific inhibitor of melatonin biosynthesis to investigate the roles of melatonin in plants.

**Figure 1:**
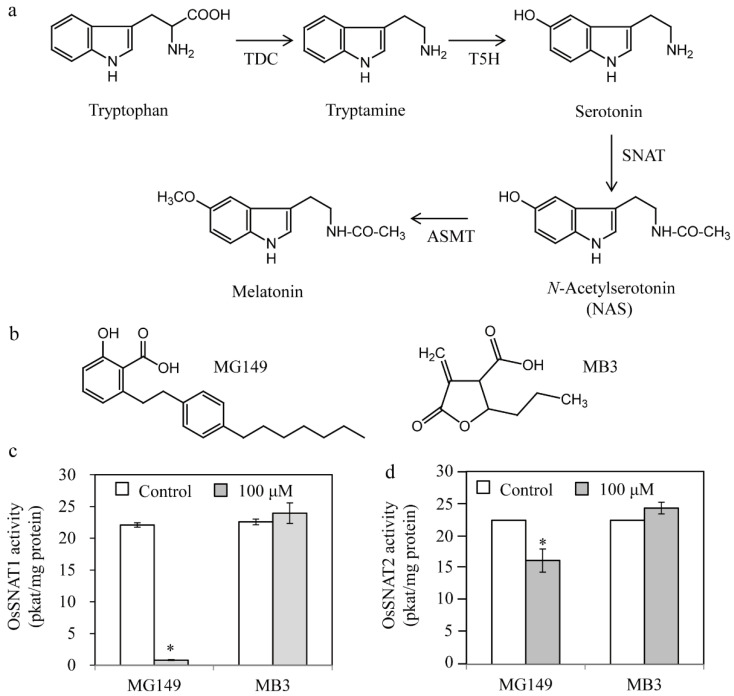
Inhibition of rice serotonin *N*-acetyltransferase by two histone acetyltransferase inhibitors. (**a**) Melatonin biosynthetic pathway with the corresponding enzymes. (**b**) Structures of the two histone acetyltransferase inhibitors. Inhibitory effects of MG149 and MB3 on rice recombinant (**c**) OsSNAT1 and (**d**) OsSNAT2. The control contained 0.1% ethanol. Bars show mean activity of three assays ± SE. Asterisks (*) indicate significant differences from the control (Tukey’s HSD; *p* < 0.05). TDC, tryptophan decarboxylase; T5H, tryptamine 5-hydroxylase; SNAT, serotonin *N*-acetyltransferase; ASMT, *N*-acetylserotonin *O*-methyltransferase.

Beginning with tryptophan, melatonin synthesis requires four consecutive enzymes (Figure 1a): tryptophan decarboxylase (TDC) catalyzes the conversion of tryptophan into tryptamine; this is followed by tryptamine 5-hydroxylase (T5H) to synthesize serotonin; serotonin *N*-acetyltransferase (SNAT) converts serotonin into *N*-acetylserotonin; and finally, *N*-acetylserotonin *O*-methyltransferase (ASMT) converts *N*-acetylserotonin into melatonin [3]. There exists many target enzymes that inhibit melatonin synthesis in plants. For example, p450 enzyme inhibitors can efficiently inhibit T5H, but p450 inhibitors such as uniconazole are not suitable for inhibiting melatonin levels because its inhibition is not directly coupled to melatonin levels in plants [23]. Another preferred target enzyme is SNAT, which belongs to the GNAT (GCN5-related *N*-acetyltransferase) family in the histone acetyltransferase (HAT) superfamily [24]. Several HAT inhibitors had been developed to inhibit HAT in animals [25]. However, there are no studies examining whether these HAT inhibitors can inhibit SNAT enzymes of either animals or plants. As an initial approach to find a chemical inhibitor of melatonin synthesis in plants, we chose the rice plant because the purified recombinant SNAT enzymes including OsSNAT1 and OsSNAT2 are available, and rice seedlings are the best sample to quantify melatonin unequivocally due to its higher abundance than other plant species [4]. We hypothesize that these HAT inhibitors can inhibit SNAT enzymes in plants in vitro and in vivo.

Here, we chose two HAT inhibitors MG149 and MB3 [26,27,28] to examine whether these inhibitors can inhibit plant SNAT in vitro and plant melatonin biosynthesis in vivo.

## 2. Materials and Methods

### 2.1. Plant Materials and Inhibitor Treatments

Surface-sterilized rice seeds (*Oryza sativa* cv. Dongjin) were germinated and grown on half-strength Murashige and Skoog medium (MB Cell, Seoul, Korea) in vertically oriented square polystyrene dishes (SPL Life Science, Pocheon-si, Korea) for 7 days at 28 °C under a 12 h light/dark cycle with 100 µmol/m^2^/s^1^ photosynthetic photon flux density. The 7-day-old seedlings were transferred to 50 mL conical polypropylene tubes containing 100 μM of the HAT inhibitors, incubated for 24 h, and the melatonin was quantified.

### 2.2. Cadmium Treatment

To examine melatonin induction with cadmium treatment, 7-day-old rice seedlings were challenged with 0.5 mM cadmium together with 100 μM of either MG149 or MB3 for 3 days under continuous light at 28 °C. The control contained 0.1% ethanol. The rice seedlings without roots were harvested, frozen rapidly in liquid nitrogen, and stored at −80 °C until the high-performance liquid chromatography (HPLC) analyses.

### 2.3. Chemical Compounds

MG149 (99.52% purity) was obtained from Selleckchem (Houston, TX, USA), and γ-butyrolactone (MB3; 95% purity) was purchased from Sigma-Aldrich (St. Louis, MO, USA). Both compounds were initially dissolved in 100% ethanol and used in a final concentration of 0.1% ethanol for treatments. Other chemicals such as tryptophan, tryptamine, serotonin, and *N*-acetylserotonin were obtained from Sigma-Aldrich (St. Louis, MO, USA).

### 2.4. Rice Recombinant SNAT1 and SNAT2 Enzymes

Rice recombinant SNAT1 protein was prepared from the N-terminal-83-amino acids deleted form of SNAT1 (AK059369), which was expressed in *Escherichia coli* harboring the plasmid vector pET300-∆83SNAT1, as described previously [29]. Rice recombinant SNAT2 was prepared from the N-terminal-34-amino acids deleted form of SNAT2 (AK068156) [30]. These two recombinant SNAT proteins were dissolved in 50% glycerol and stored at −20 °C until further analysis.

### 2.5. Measuring SNAT Enzyme Activity

The purified recombinant SNAT proteins were incubated in a total volume of 100 µL containing 0.5 mM serotonin and 0.5 mM acetyl-CoA in 100 mM potassium phosphate (pH 8.8) either at 55 °C (SNAT1) or at 45 °C (SNAT2) in the presence of various inhibitor concentrations to see whether the addition of inhibitors inhibit the synthesis of *N*-acetylserotonin compared to the control (absence of inhibitors). After 30-min incubation, the reaction was stopped by the addition of 20 µL acetic acid and 30 µL methanol. A 10 µL aliquot was subjected to high-performance liquid chromatography (HPLC) using a fluorescence detector system, as described previously [31]. Protein concentrations were determined using the Bradford method and a protein assay dye (Bio-Rad, Hercules, CA, USA). The analysis was performed in triplicate.

### 2.6. Melatonin Quantification

Frozen rice seedlings (0.1 g samples) were pulverized to a powder in liquid nitrogen using a TissueLyser II (QIAGEN, Tokyo, Japan) and extracted with 1 mL of chloroform for 1 h at room temperature. The chloroform extracts (200 µL) were evaporated completely and dissolved in 0.1 mL of 40% methanol; 10 µL aliquots were subjected to HPLC using a fluorescence detector system (Waters, Milford, MA, USA). Melatonin was detected at 280 nm (excitation) and 348 nm (emission), as described previously [31]. All measurements were made in triplicate.

### 2.7. Quantification of Tryptophan, Tryptamine, Serotonin, and N-Acetylserotonin

The powder was extracted with 1 mL of methanol followed by HPLC analysis in an isocratic elution with 6% ethanol in 0.3% trifluoroacetic acid for 50 min at a flow rate of 1 mL/min. Compounds were detected at 280 nm (excitation) and 348 nm (emission).

### 2.8. Laminar Joint Inclination Assay

The lamina inclination assay was performed, as described previously [32]. Rice was grown in soil in a culture room at 28 °C for 7 days under a 12 h light/12 h dark photoperiod. Approximately 2-cm-long second leaf lamina joints were floated on 0.1% ethanol containing either MG149 or MB3 for 3 days in the dark at 28 °C, and the lamina joint angle of the second leaf was measured.

### 2.9. Statistical Analyses

Asterisks indicate significantly different values at *p* < 0.05, according to post hoc Tukey’s honestly significant difference (HSD) tests. Data are presented as means ± standard deviation.

## 3. Results

### 3.1. In Vitro Inhibition of Rice SNAT Enzymes by HAT Inhibitors

**Figure 2:**
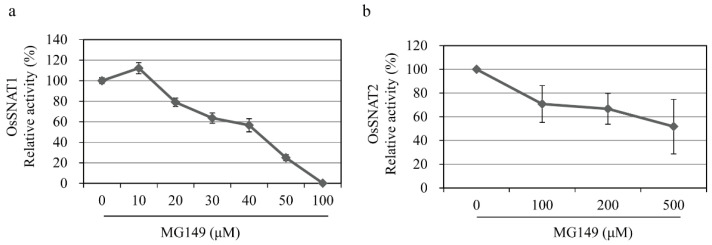
Inhibition of SNAT activity by varying concentrations of inhibitors. Dose-dependent inhibition of rice recombinant (**a**) SNAT1 (OsSNAT1) and (**b**) SNAT2 (OsSNAT2) by different concentrations of MG149. The assays were performed in the assay buffer containing 0.1% ethanol.

The HAT inhibitors MG149 and MB3 were chosen based on reports of their use for HAT inhibition in plants [26,28,33] (Figure 1b). MG149 inhibits HAT by competitively binding the acetyl-CoA binding site, while MB3 binds the active sites of HAT proteins [26,34]. The rice recombinant SNAT proteins OsSNAT1 and OsSNAT2 were used to examine whether the HAT inhibitors can inhibit plant SNAT proteins in vitro. As shown in Figure 1c, OsSNAT1 activity was abolished in the presence of 100 μM MG149, whereas the OsSNAT2 activity decreased by 28%. By contrast, MB3 had no effects on either SNAT enzyme (Figure 1c,d). These results suggest that MB3, which is commonly used to inhibit plant HAT, is not associated with the inhibition of plant melatonin biosynthesis, while MG149 is a potent melatonin synthesis inhibitor. To examine whether MG149 inhibits SNAT activity in a dose-dependent manner, the relative SNAT activity was measured in the presence of various MG149 concentrations (Figure 2). The relative SNAT1 activity decreased by 20% in 20 μM MG149 and by 80% in 50 μM MG149. The degree of SNAT2 inhibition with MG149 was moderate in comparison. These results clearly indicate that MG149 inhibits both rice SNAT isoforms in vitro.

### 3.2. In Vivo Inhibition of Melatonin Synthesis by HAT Inhibitors

**Figure 3:**
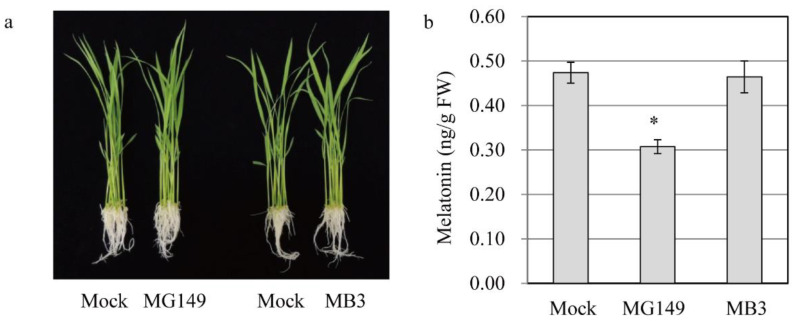
Quantification of melatonin in rice seedlings in response to MG149 and MB3. (**a**) Photograph of rice seedlings treated with inhibitors for 24 h. (**b**) Melatonin content in 7-day-old rice seedlings rhizosperically treated with 100 μM inhibitor for 24 h and subjected to HPLC analysis for melatonin quantification. The mock contained 0.1% ethanol. Asterisk (*) indicates significant difference from the mock control (Tukey’s HSD; *p* < 0.05).

To examine whether the in vitro inhibition of SNAT activity by MG149 is coupled functionally with the inhibition of melatonin synthesis in vivo, we examined 7-day-old rice seedlings that were challenged rhizosperically with either MG149 or MB3 for 24 h and quantified the melatonin. There were no phenotypic changes between mock and treatments, indicative of no toxic effects of HAT inhibitors (Figure 3a). The control containing 0.1% ethanol produced 0.46 ng/g FW melatonin, whereas the melatonin production decreased to 0.3 ng/g FW with 100 μM MG149, suggesting that MG149 efficiently inhibits melatonin production by inhibiting SNAT (Figure 3b). However, in vivo MB3 treatment produced melatonin comparable to the control, consistent with the in vitro result.

### 3.3. Quantification of Melatonin and Its Precursors with Cadmium Treatment

**Figure 4:**
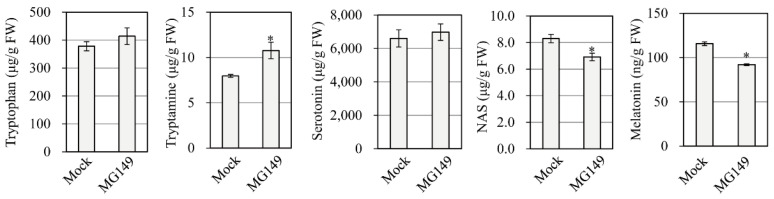
Quantification of melatonin and its precursors in rice seedlings. The 7-day-old rice seedlings were rhizosperically treated with 100 μM MG149 plus 0.5 mM cadmium for melatonin induction for 3 days. The mock contained 0.1% ethanol. Asterisks (*) indicate significant differences from the mock control (Tukey’s HSD; *p* < 0.05).

Melatonin synthesis is induced in response to cadmium treatment in rice, which facilitates the quantification of melatonin and its precursors [35]. To confirm that melatonin levels were reduced in healthy rice seedlings challenged with 100 μM MG149, 7-day-old rice seedlings were challenged with 100 μM MG149 plus 0.5 mM cadmium and incubated for 3 d at 28 °C under a continuous light regime. As shown in Figure 4, cadmium treatment alone produced 112 ng/g FW melatonin, whereas cadmium plus MG149 resulted in a 20% decrease in melatonin synthesis over cadmium alone. *N*-Acetylserotonin, a direct product of the SNAT reaction, was decreased by 13% in the MG149-treated seedlings, whereas other melatonin precursors, such as tryptophan and serotonin, did not differ between treatments. The increase in tryptamine with MG149 seemed to be an elicitor effect of MG149 because TDC is induced in response to a diverse array of biotic and abiotic stresses [36]. The in vivo SNAT inhibitory effect of MG149 in rice seedlings is too low compared to that of in vitro data, resulting in a modest 20% decrease in melatonin synthesis. These data suggest that the suppression of SNAT1 is not sufficient to abolish melatonin synthesis in plants possibly due to the presence of SNAT2.

### 3.4. Measuring the Response of the Second Leaf Lamina Joint Angle to MG149

**Figure 5:**
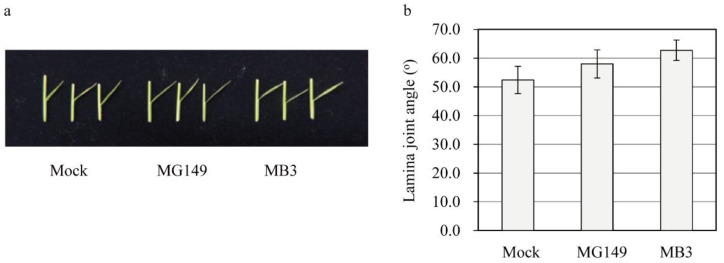
Measuring the lamina joint angle in response to MG149 and MB3. (**a**) Photograph of the lamina joint angle. (**b**) Bending angle of lamina joint. Seven-day-old rice seedlings grown in soil were dissected at the second leaf lamina joint and floated on mock solution containing either MG149 or MB3 for 3 days in the dark. The mock contained 0.1% ethanol.

Decreased melatonin in rice results in an erect leaf phenotype via decreased brassinosteroid (BR) synthesis due to the reduced expression of DWARF4, a rate-limiting BR biosynthesis gene [7]. To assess whether the MG149-mediated melatonin decrease is associated with the physiological function of melatonin in rice, second leaf lamina joints of the 7-day-old seedlings grown in soil were floated on the mock solution containing 100 μM of either MG149 or MB3 for 3 days in the dark before measuring the degree of lamina inclination. Neither MG149 nor MB3 led to a decrease in the lamina angle (Figure 5). The main reason for this is ascribed to the insufficient suppression of SNAT2 activity by MG149, because SNAT2-suppressed melatonin synthesis plays a determining role in the lamina angle decrease via the BR decrease [37].

## 4. Discussion

Plant and animal SNAT proteins belong to the GNAT family and share a conserved acetyl CoA binding domain and acetyl transfer mechanism [38]. Although no significant amino acid identity between plant and animal SNAT was observed, both are small ca. 20,000 kDa proteins, while the HAT proteins GCN5 [26], p300 [24], and Tip60 [39] have a molecular weight greater than 50,000 kDa. Moreover, animal SNAT protein is located in mitochondria, whereas plant SNATs are localized in chloroplasts. Another major difference is that animal SNAT is mainly regulated by protein phosphorylation, but plant SNATs do not possess any protein phosphorylation sites [3].

SNAT proteins from animals are inhibited by bisubstrate HAT inhibitors and many natural products [24]. A representative bisubstrate for sheep SNAT is coenzyme A-S-acetyltryptamine, which is a competitive acetyl-CoA inhibitor and a noncompetitive tryptamine inhibitor with an IC_50_ of about 150 nM [40]. One major drawback of this bisubstrate inhibitor is that it is not very cell permeable [24]. The natural product flavonoid myricetin was discovered to inhibit SNAT enzyme activity in rat pineal gland extracts with an IC_50_ of 1.64 μM [41]. In addition, the flavonoids quercetin, myricetin, and morin significantly inhibited sheep SNAT activity and rice recombinant SNAT enzymes [42]. The natural product anacardic acid and its derivatives also inhibit HAT proteins such as p300 and Tip60 [25,34]. Recently, the anacardic acid derivative MG149 was used to inhibit HAT in both animals and plants [27,28,34]. However, there are no reports on the possible relationship between HAT inhibitors and melatonin biosynthesis in plants. Loss-of-function analyses, such as knockout and RNA interference (RNAi), have been used extensively to investigate the functional roles of melatonin in model plants, such as Arabidopsis and rice [43,44]. As an alternative, the discovery of chemical inhibitors of melatonin biosynthesis in plants will provide an effective approach to examine the roles of melatonin in plants that are not accessible using transgenic approaches. Here, we found that the HAT inhibitor MG149 efficiently inhibited the activity of rice recombinant SNAT1 in vitro but moderately inhibited SNAT2. MG149 treatment decreased melatonin synthesis in rice seedlings that were treated rhizosperically. However, no dramatic inhibition of melatonin synthesis was achieved in rice seedlings with MG149 due to its moderate inhibition of SNAT2. MG149 is a competitive inhibitor for acetyl-CoA and its binding kinetics vary among protein substrates [34]. A possible explanation for insufficient inhibition of MG149 against OsSNAT2 is that its binding in the acetyl-CoA binding pocket of OsSNAT2 may not be higher than that of OsSNAT1 due to the low amino acid identity, with about 39% between OsSNAT1 and OsSNAT2 isoenzymes [29,30]. MB3 requires specific amino acid sites of EXXXC for its inhibition. Although OsSNAT1 and OsSNAT2 possess similar motifs, such as DXXXXC and EXXC, respectively, MB3 was unable to recognize their sites. Thus, MB3 treatment acts like the control that does not inhibit OsSNATs. We plan to conduct further studies of plant SNAT inhibitors that will greatly alter melatonin levels in plants.
